# Machine-Learning-Based Near-Surface Ozone Forecasting Model with Planetary Boundary Layer Information

**DOI:** 10.3390/s22207864

**Published:** 2022-10-16

**Authors:** Kabseok Ko, Seokheon Cho, Ramesh R. Rao

**Affiliations:** 1 Department of Electronics Engineering, Kangwon National University, Chuncheon 24341, Korea; 2 Qualcomm Institute, University of California, San Diego (UCSD), San Diego, CA 92093, USA

**Keywords:** ozone forecasts, machine learning, numerical weather prediction, planetary boundary layer, LSTM, MLP

## Abstract

Surface ozone is one of six air pollutants designated as harmful by National Ambient Air Quality Standards because it can adversely impact human health and the environment. Thus, ozone forecasting is a critical task that can help people avoid dangerously high ozone concentrations. Conventional numerical approaches, as well as data-driven forecasting approaches, have been studied for ozone forecasting. Data-driven forecasting models, in particular, have gained momentum with the introduction of machine learning advancements. We consider planetary boundary layer (PBL) height as a new input feature for data-driven ozone forecasting models. PBL has been shown to impact ozone concentrations, making it an important factor in ozone forecasts. In this paper, we investigate the effectiveness of utilization of PBL height on the performance of surface ozone forecasts. We present both surface ozone forecasting models, based on multilayer perceptron (MLP) and bidirectional long short-term memory (LSTM) models. These two models forecast hourly ozone concentrations for an upcoming 24-h period using two types of input data, such as measurement data and PBL height. We consider the predicted values of PBL height obtained from the weather research and forecasting (WRF) model, since it is difficult to gather actual PBL measurements. We evaluate two ozone forecasting models in terms of index of agreement (IOA), mean absolute error (MAE), and root mean square error (RMSE). Results showed that the MLP-based and bidirectional LSTM-based models yielded lower MAE and RMSE when considering forecasted PBL height, but there was no significant changes in IOA when compared with models in which no forecasted PBL data were used. This result suggests that utilizing forecasted PBL height can improve the forecasting performance of data-driven prediction models for surface ozone concentrations.

## 1. Introduction

Surface ozone is one of six air pollutants that have a safety level set by National Ambient Air Quality Standards, since it can adversely impact human health and the environment. Short-term exposure to ozone is related to mortality in urban countries [1]; for example, a 0.45% increase in mortality was attributed to a 10 ppb increase in ozone [2]. To protect human beings from these harmful effects, the U.S. Environmental Protection Agency (EPA) set a target value of maximum ozone concentrations to an eight-hour average concentration of 70 ppb and 120 ppb within a day and a calendar year, respectively. U.S. environmental policies for ozone reduction are made and implemented based on these values. While these policies can help reduce overall surface ozone pollution, daily ozone levels are not always lower than the suggested values. As a complementary measure, future ozone information could be provided to the public to help with individual decision making and provide warnings to avoid events of high ozone concentrations. Hence, ozone forecasting is a critical task with high potential impact.

Ozone forecasts can be conducted using numerical methods and/or data-driven approaches. Numerical methods provide predictions of ozone concentrations by simulating emissions, chemistry, physics of the atmosphere, and chemical transformation and transportation processes. For numerical methods, there are two well-known forecasting models: the Community Multiscale Air Quality (CMAQ) model developed by the U.S. EPA [3] and the Weather Research and Forecasting/Chemistry (WRF-chem) model [4]. Both of these methods need substantial computational time and have limited accuracy due to use of simplified assumptions in the fields of physics and chemistry. In addition, ozone concentrations at local spots may be different from ozone forecasts by numerical methods because of limited spatial resolution.

Data-driven approaches forecast ozone concentrations based on data obtained from measurement stations. Compared to numerical methods, these approaches can predict ozone concentrations with less computational burden as well as at microscopic scales. Data-driven approaches can be categorized into statistical and machine learning methods. Statistical methods utilize statistics to predict ozone concentrations. Some examples of statistical methods are autoregressive integrated moving average and multiple linear regression. Su et al. [5] evaluated several statistical methods for hourly ozone concentration prediction, including kernel extreme learning machine (KELM), support vector machine regression, back propagation neural network, and stepwise regression. As machine learning techniques have advanced, many studies have applied machine-learning algorithms, e.g., convolutional neural network (CNN) and long short-term memory (LSTM) networks to predict ozone concentrations [6,7,8]. Freeman et al. [9] presented a recurrent neural network with LSTM to forecast averages of 8-hour surface ozone concentrations. Jia et al. [10] employed a sequence-to-sequence deep learning model, which has a gated recurrent unit (GRU)-based encoding forecasting structure, to forecast up to the next 6 h of ozone concentrations over the Yangtze River Delta region of eastern China. Eslami et al. [6] used a deep convolutional neural network (CNN) to forecast hourly ozone concentrations for a lead time of 24 h based on chemical and meteorological measurement data obtained from Seoul, South Korea. An identified limitation of deep CNN models is that the models do not provide good performance during cold months or under the ambient environment with high ozone concentrations [7]. Ma et al. [11] proposed a stacked bidirectional LSTM with a transfer learning to predict three air pollutants, such as ozone, nitro-dioxide, and particulate matter with a diameter of less than 2.5 microns. Kleinert et al. [8] applied multiple CNN layers to forecast the daily maximum 8-h average ozone concentrations for up to 4 days.

The selection of input features used in data-driven approaches is one of the most important tasks for improving forecast accuracy. Many studies have selected input features according to revealed mechanisms of ozone formation as well as atmosphere physics. Near-surface ozone is generated from the oxidation of volatile organic compounds (VOCs) and nitrogen oxides (NO${}_{x}$) under sunlight [12]. Many studies have considered chemical (O${}_{3}$, NO, and NO${}_{2}$) and meteorological measurement data (temperature, relative humidity, wind direction, and wind speed) as input features.

The planetary boundary layer (PBL), which determines vertical transports of heat, water vapor, and aerosols between the underlying surface and atmosphere, has been shown to impact air pollutants [13,14], including ozone. Some studies have recently shown that the fate of air pollutants is impacted by the PBL [14,15]. The mixing of the PBL can result in diurnal cycles in air pollutant concentrations [16], and it was shown that stable weather conditions can promote elevations in ozone concentrations. Another study showed that the ozone in the residual layer is downward transported to the PBL as PBL height increases [17]. The difference in PBL height on days between high ozone and low ozone concentrations is not significant, but the PBL growth rate is related to ozone concentrations [18]. Like these studies, PBL height could be an important factor in ozone concentration predictions in addition to meteorological parameters. Many studies on ozone forecasting have applied machine learning and deep learning algorithms to forecast ozone concentrations, but the use of the forecasted values of PBL height as an input feature in these algorithms has not been considered.

In this paper, we investigate the effectiveness of integrating PBL height into data-driven ozone forecasting models on forecast accuracy. Since PBL height, as well as meteorological parameters, have a nonlinear relationship with ozone concentrations, we employ machine learning algorithms that are capable of modeling nonlinearity between input and output, i.e., multilayer perception (MLP) and bidirectional long short-term memory (LSTM). We present two ozone forecasting models based on two machine learning algorithms where ozone, temperature, and relative humidity data, as well as the PBL height data, are used as input features. We consider the predicted PBL height for two forecasting models, instead of their real measurements, since it is difficult to gather actual PBL data. Two types of inputs, such as the measurement and predicted data, have a sequential relation in time and a different number of features, i.e., the number of features for measurement data and PBL height are 3 and 1, respectively. We apply different techniques for integrating two different types of inputs in two ozone forecasting models. In the case of bidirectional LSTMs, we present two separate bidirectional LSTMs where each LSTM is connected sequentially and each one is individually connected to each of two inputs. We evaluate the performance of the two ozone forecasting models and verify the effectiveness of using PBL height in the forecasting models.

The rest of the paper is organized as follows: we describe the background of MLP and bidirectional LSTM models in Section 2. In Section 3, the MLP and bidirectional LSTM models for ozone prediction are described. In Section 4, we evaluate the performance of the models by comparing with conventional models. Finally, we draw conclusions in Section 5.

## 2. Preliminaries: MLP and Bidirectional LSTM

In this section, we describe the multilayer perceptron (MLP) and the bidirectional LSTM networks, which have been widely used for time-series prediction problems.

### 2.1. Multilayer Perceptron (MLP)

An artificial neural network (ANN) is a powerful tool for approximating complex non-linear relationships between input and output based on a collection of connected units or nodes. Concepts of the units or nodes and their connections are inspired by the behavior of the human brain and nervous systems. Based on ANN, various models have been proposed and implemented for classification and sequential analysis. The MLP, a class of feedforward neural network, is one of the most widely used ANN-based models.

MLP is comprised of an input layer, one or more hidden layers, and an output layer. The layers have neurons, each of which is connected to neurons in adjacent layers. Each connection is specified by adaptable weights. Each neuron receives weighted input signals from outputs of other neurons. Each neuron produces an output, which is a function of the weighted input, bias, and an activation function. This is expressed as follows:

(1) $$\begin{array}{c} y=f(\sum w_{i}x_{i}+b), \end{array}$$

where *y* is a neuron output, $x_{i}$ is the *i*-th input to the neuron, $w_{i}$ is a connection weight of the *i*-th input, *b* is a bias, and *f* is an activation function. Examples of activation functions are sigmoids and hyperbolic tangents.

### 2.2. Long Short-Term Memory

**Figure 1:**
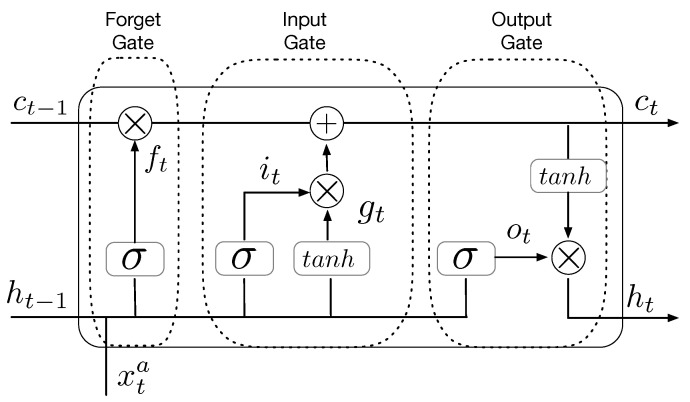
Architecture of an LSTM cell, including forget, input, and output gates.

As a class of ANN-based neural networks, recurrent neural networks (RNN) have been used for sequential data or time-series data. The traditional RNN models have the vanishing gradient problem, which makes the establishment of long-term temporal relationships difficult. To overcome this limitation of RNN models, an LSTM cell was proposed in 1997 by Hochreiter and Schmidhuber [19] and was later modified by Gers, Schmidhuber, and Cummins in 1999 [20]. One of the widely used LSTM architectures is shown in Figure 1. The LSTM architecture has three types of control gates: forget, input, and output. These control gates are the distinct difference between a traditional RNN and an LSTM network. Hence, the LSTM model has been the most successful model and has received a lot of attention in many applications, e.g., classification [21] and time-series forecasting [22,23,24].

Figure 1 shows basic units of an LSTM cell, such as input node $g_{t}$, input gate $i_{t}$, forget gate $f_{t}$, and output gate $o_{t}$, as well as the connections among units. Referring to the connections, all nodes in an LSTM cell can be mathematically expressed as follows:

(2) $$\begin{array}{cc} f_{t} & =\sigma (W_{fx}x_{t}+W_{fh}h_{t-1}+b_{f}) \end{array}$$

(3) $$\begin{array}{cc} i_{t} & =\sigma (W_{ix}x_{t}+W_{ih}h_{t-1}+b_{i}) \end{array}$$

(4) $$\begin{array}{cc} g_{t} & =\phi (W_{gx}x_{t}+W_{gh}h_{t-1}+b_{g}) \end{array}$$

(5) $$\begin{array}{cc} o_{t} & =\sigma (W_{ox}x_{t}+W_{oh}h_{t-1}+b_{o}) \end{array}$$

(6) $$\begin{array}{cc} c_{t} & =g_{t}⊙i_{t}+c_{t-1}⊙f_{t} \end{array}$$

(7) $$\begin{array}{cc} h_{t} & =\phi \left(s_{t}\right)⊙o_{t}, \end{array}$$

where $W_{fx},W_{fh},W_{ix},W_{ih},W_{gx},W_{gh},W_{ox},$ and $W_{oh}$ are weight matrices for the corresponding inputs of the network activation functions. $b_{f},b_{i},b_{g},$ and $b_{o}$ are bias values. ⊙ denotes an element-wise multiplication. σ and ϕ denote the sigmoid and tanh activation functions, respectively.

In addition to these nodes, the LSTM architecture has an important state called a memory cell state ($c_{t}$), which has the function of establishing temporal patterns. The memory cell state $c_{t}$ is updated based on outputs of two gates, such as the forget and input gates. The forget gate can determine what information on the memory cell state will be erased. For example, when the forget gate has a value of 0, information is erased. On the other hand, information is kept when the gate has a value of 1. The input gate can control what information from the input node will be kept.

### 2.3. Bidirectional LSTM

**Figure 2:**
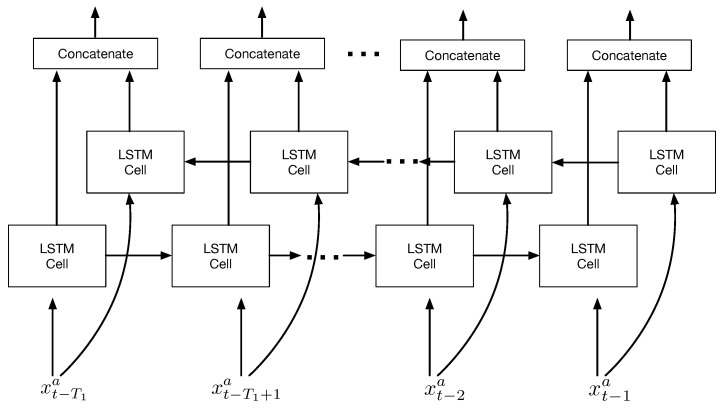
Architecture of a bidirectional LSTM network.

The idea of bidirection was first proposed in RNNs, which can only make use of forward dependencies, in order to exploit backward dependencies [25]. To overcome the problem of long-term dependency in bidirectional RNNs, bidirectional LSTM networks were proposed [26]. There are two separate LSTM layers in bidirectional LSTM networks, each of which can capture both forward and backward dependencies in sequence data. Two outputs of LSTM layers are connected to the same output layer. With this capability, bidirectional LSTM can make better predictions in sequential prediction tasks by exploiting long-term dependencies [26,27,28]. Figure 2 shows an example of a bidirectional LSTM network where two parallel LSTM layers are put in place of forward and backward directions.

## 3. Ozone Forecasting Models

Conventional data-driven ozone prediction models have used historical ozone data and other historical parameters, such as temperature, pressure, relative humidity, wind speed, wind direction, and NOx as input features. These models have extracted relationships between past measurement data and future ozone concentrations so that forecasting ozone concentrations can be fulfilled based on its measurement data.

In the area of air quality research, attention has recently been paid to the planetary boundary layer (PBL) as a meteorological parameter that determines the vertical transport of heat, water vapor, and aerosols between the underlying surface and atmosphere. The PBL characteristics have an impact on the fate of air pollutants [14,15]. For example, diurnal cycles of ozone concentration can result from the mixing of the PBL [16], and stable weather conditions can be favorable for elevating ozone concentrations. As PBL height increases, the ozone in the residual layer is downward transported to the PBL [17]. The difference in PBL height on days between high ozone and low ozone concentrations is not significant, but the PBL growth rate is related to ozone concentrations [18]. PBL height can be an important factor in ozone concentration predictions in addition to meteorological parameters, such as temperature, relative humidity, wind speed, and wind direction. Thus, we consider PBL height in ozone forecasting models as a new input feature.

In the following sections, we present two different types of ozone forecasting models, each of which is based on MLP and bidirectional LSTM models, respectively. We use forecast data for PBL heights as a new input feature to two ozone forecasting models because it is difficult to gather actual measured PBL heights. The predicted PBL height data have different dimensions of time from the measurement data used for other input features, so they cannot be directly applied. We present different techniques for using two different types of inputs in two ozone forecasting models.

### 3.1. Mlp-Based Forecasting Model

We forecast ozone concentrations for $T_{2}$ hours ahead using the past data of ozone, temperature, and relative humidity with $T_{1}$ hours, as well as the predicted PBL heights for future $T_{2}$ hours. The predicted PBL heights can be obtained from WRF models. To use two different inputs in an MLP-based ozone prediction model, we need a structure, since the past measurement data and the predicted PBL heights have different dimensions, i.e., the past measurement input and the predicted PBL heights have $T_{1}\times 3$ and $T_{2}\times 1$ dimensions, respectively. These inputs cannot be directly concatenated to apply to MLP models. Therefore, we make the past measurement and predicted PBL height data flattened and concatenated, and then these combined data are used as an input to MLP models. For hidden layers, we consider one or two layers. An output layer generates $T_{2}$-hour ozone forecasts. In this study, we set all activation functions of layers to hyper-tangents.

Ozone, temperature, and relative humidity, which are used as past measurement inputs in our ozone prediction models, can be measured at sensor stations, but the predicted PBL heights cannot be obtained from the stations. Alternatively, the forecasted value of the PBL heights is available with the help of many WRF models, which generate short-term and medium-term forecasts in different intervals. We focus on the WRF model that can generate forecasted values of the PBL heights every hour for more than the next 24 h, which will allow us to use the PBL height data in our proposed ozone prediction models. For example, the North America Mesoscale (NAM) model in the United States forecasts regional weather parameters, including PBL heights, every hour up to 36 h. The forecasts are issued every 6 h. We note that our ozone prediction models have a limit on prediction time horizon due to the data availability of predicted PBL heights.

### 3.2. Bidirectional LSTM-Based Forecasting Model

Similar to the MLP-based ozone forecasting model, two different types of inputs are employed in our bidirectional LSTM-based ozone prediction model. One type is the measurement data of ozone, temperature, and relative humidity. The other one is the predicted PBL heights obtained from the WRF model. The two inputs have a sequential relation in time, but have different time dimensions, i.e., $T_{1}\times 3$ for the measurement data and $T_{2}\times 1$ for the predicted PBL height data. Concatenating two inputs over time cannot be applied to a single bidirectional LSTM network due to different dimensions because input to a single bidirectional LSTM network must have the same dimension over time. Thus, we need a sequential bidirectional LSTM model where two bidirectional LSTM models are connected to each other.

**Figure 3:**
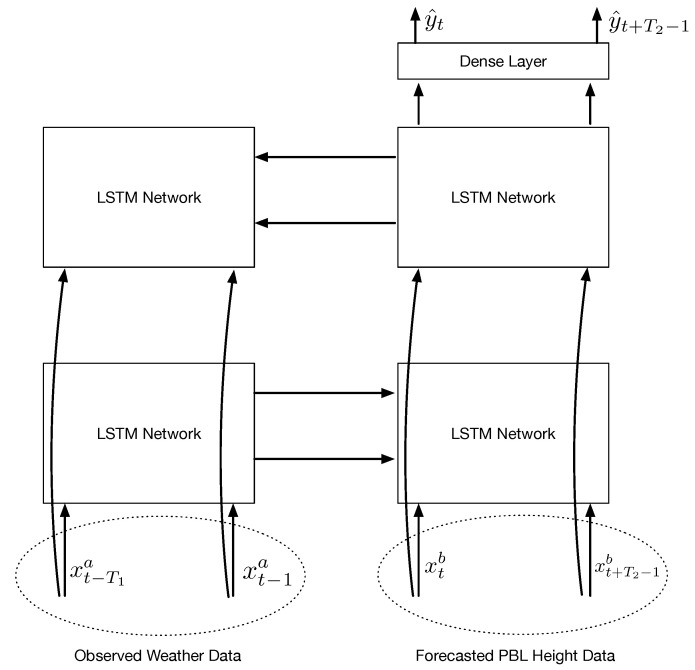
Proposed ozone forecast model based on the bidirectional LSTM structure.

Figure 3 shows the bidirectional LSTM-based ozone prediction model. This model has a sequential structure in LSTM networks in forward and backward directions. LSTM networks in forward and backward directions are placed on the lower and upper layers in Figure 3. In the proposed model, the left LSTM networks at the lower and upper layers use the measurement data, such as ozone, temperature, and relative humidity as inputs. The right ones use the predicted PBL heights as an input. The left LSTM networks learn an appropriate latent representation from the observed ozone, temperature, and relative humidity. The latent representation and PBL heights are used to reconstruct ozone concentrations through the right LSTM networks. The right LSTM networks on both of the layers generate outputs with the recurrent sequence set to be true. The two outputs provided from the two right-side LSTM networks are concatenated in a time-distributed manner, and then the $T_{2}$ concatenated outputs are connected to $T_{2}$ dense layers at the output layer. Each of the $T_{2}$ dense layers generates predicted ozone concentrations at the corresponding time for the next $T_{2}$ hours. We note that when we concatenate two LSTM networks, we set the dimensions of outputs in two LSTMs to being equal so that the memory cell state in the left LSTM can be transferred to the right one.

The four LSTM networks have the same activation function of hyper-tangent and the same recurrent activation function of the sigmoid in this paper. The output layer has an activation function of hyper-tangents.

## 4. Experiments

In this section, we compare the performance of the MLP-based and bidirectional LSTM-based ozone forecasting models and evaluate the effectiveness of the use of the predicted PBL heights on accuracy improvement. To this end, ozone data collected in California, USA are used in this paper.

### 4.1. Data Sets

#### 4.1.1. Numerical Weather Data

Our ozone forecasting models need the predicted $T_{2}$-hour PBL height data. The predicted PBL height data can be provided by WRF models. In this paper, we select the North American Mesoscale (NAM) model, which is one of the regional weather forecast models run by the National Centers for Environmental Prediction (NCEP), as a WRF model. The NAM model forecasts several weather parameters, such as temperature, relative humidity, wind direction, wind speed, and PBL heights over the North American continent four times daily at times 00z, 06z, 12z, and 18z over grid points with a 12 km horizontal resolution. The weather forecasts are generated with a one-hour interval up to the next 36 h and a three-hour interval until the next 84 h.

The NAM data set can be downloaded from the Archive Information Request (AIR) systems of the National Centers for Environmental Information. It is written in a grib2 format, which is a file format for gridded meteorological data. We collect NAM data from 1 January 2017 to 31 December 2019. We extract PBL height forecasts at specific grid points from the collected NAM data set to make training and test sets for our proposed ozone forecasting models. The grid points used to forecast PBL heights are related to the locations of ozone monitoring stations.

#### 4.1.2. Measured Ozone, Temperature, and Relative Humidity Data

In the United States, the EPA operates air quality monitoring stations that measure six criteria pollutants, including ozone. We can download historical ozone data collected by these stations, including hourly ozone concentrations, using the EPA API. Meteorological parameters, such as temperature and relative humidity, are also measured by the stations and can be downloaded using the EAP API.

**Figure 4:**
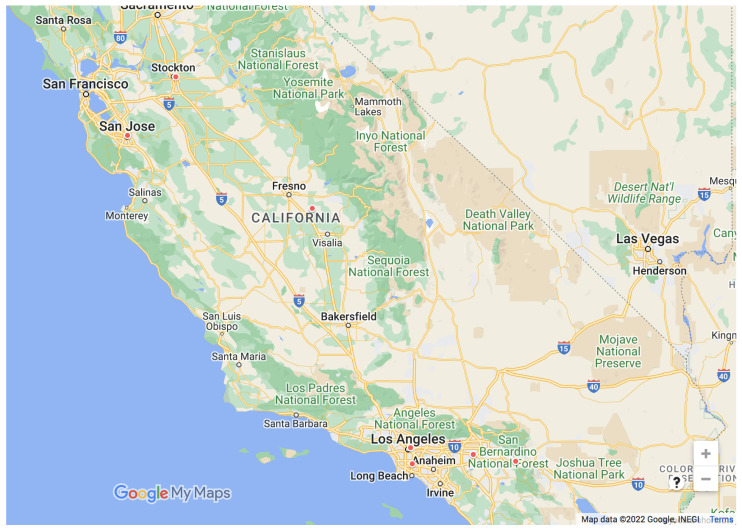
Location of the considered ozone monitoring stations.

We collect the historical ozone, temperature, and relative humidity data from 1 January 2017 to 31 December 2019. Table 1 lists the GPS information and location names of the EPA monitoring stations considered in this paper, and Figure 4 shows the location of the considered air quality monitoring stations. Since our ozone forecasting models use predicted rather than actual PBL heights, we need to determine the appropriate grid points used to obtain the predicted PBL heights. We select one of the grid points close to each corresponding EPA monitoring station. Table 2 shows the latitude and longitude of the selected grid point corresponding to each air quality monitoring station, as well as the distance in km to its corresponding air quality monitoring station.

### 4.2. Data Preprocessing

We perform max-min normalization to ozone, temperature, relative humidity, and PBL height to normalize their values having different scales. The normalized values are re-scaled in values from −1 to 1.

The data set measured at the monitoring stations and provided from the NAM model has missing values. For measurement data at air quality monitoring stations, linear imputation is applied to missing values when they are not continuous. In the case of the NAM data set, some data during 2017 to 2019 is missing, but no imputation is applied.

Each sample is considered for making the training and test sets for our proposed ozone forecasting models, whenever both data from the monitoring stations and the NAM model have no missing values for $T_{1}$ and $T_{2}$ hours, respectively. In other words, we remove samples, including missing values on the monitoring stations or NAM data. For the training set, each sample is generated by a 1-h sliding move during 2017. On the other hand, a 24-h sliding move is applied to evaluate the daily average performance on the test set from 2018 to 2019.

### 4.3. Evaluation Metrics

We employ mean absolute error (MAE), root mean square error (RMSE), and index of agreement (IOA) to evaluate the proposed ozone prediction models and verify the effectiveness of using PBL heights as an input on the ozone prediction performance. MAE and RMSE represent the amount of error between observed and predicted values, which are the most widely used performance metrics in many research areas, including ozone forecasting. The IOA, which was proposed by Willmott [29], has been used to evaluate how well a predicted sequence fits to an observed sequence, and its value varies between 0 and 1. When two sequences are perfectly matched, IOA is equal to 1. IOA of 0 means that two sequences have no agreement with each other. The MAE, RMSE, and IOA are mathematically expressed as follows:

(8) $$\begin{array}{cc} \; & IOA=1-\frac{\sum_{i=1}^{n}{\left|X_{i}-Y_{i}\right|}^{2}}{\sum_{i=1}^{n}\left(\right|X_{i}-\bar{X}\left|+\right|Y_{i}-\bar{X}{\left|\right)}^{2}}, \end{array}$$

(9) $$\begin{array}{cc} \; & MAE=\frac{\sum_{i=1}^{n}\left|X_{i}-Y_{i}\right|}{n}, \end{array}$$

(10) $$\begin{array}{cc} \; & RMSE=\frac{\sum_{i=1}^{n}{\left(X_{i}-Y_{i}\right)}^{2}}{n}, \end{array}$$

where $X_{i}$ and $Y_{i}$ represent the observed and the forecasted values at *i*-th time, respectively, and $\bar{X}$ denotes the mean value of $\left\{X_{1},X_{2},\ldots ,X_{n}\right\}$.

### 4.4. Simulation Results

We evaluate the impact of utilization of the forecasted PBL heights on ozone forecasting performance. We compare performances of MLP-based and bidirectional LSTM-based forecasting models in terms of IOA, MAE, and RMSE with the following two cases: (1) using only the historical meteorological measurement data and (2) both the historical meteorological measurement data and the forecasted PBL heights.

For MLP-based ozone forecast models, we consider a single and two hidden layers regardless of use of the predicted PBL heights. We set the activation function at all layers, such as input, hidden, and output layers, to a sigmoid function. There are several hyperparameters in MLP models, i.e., the number of nodes in input and hidden layers, to be determined. We perform a grid search to obtain the best forecasting performance. For the number of nodes in the input layer and hidden layers, search ranges are set to {8,16,24,32,64} and {8,16,24,32,48,64,96,128,256}, respectively. We considered a range of the number of nodes in the input layer as {8,16,24,32,48,64,96,128,256} initially, but we reduced the search range since higher values showed worse performance. We also consider several epochs of {50,100,150,200,250,300,350,400}.

For bidirectional LSTM-based models, we need to set up the dimension of outputs at all LSTM blocks. We note that all LSTM networks are set to have the same dimension. To obtain the best performance of ozone forecasts, a grid search is also applied for a set of {4,8,16,24,32,48,64} and several epochs of {50,100,150,200,250,300,350,400}.

Table 3 shows the best performance of the MLP- and LSTM-based surface ozone forecasting models with and without predicted PBL heights in terms of IOA, MAE, and RMSE. It is shown that MAE and RMSE for all stations decrease as the number of hidden layers in MLP models increases. Decreases in MAE for all stations are from 0.12% to 1.35%. Considering that it requires a lot of computation in hyperparameter optimization, increasing the number of hidden layers seems to be not necessarily a good thing.

Utilization of the forecasted PBL heights at the forecast horizon improves MAE and RMSE for both the MLP-based and bidirectional LSTM-based models. In the case of station 6-065-8001, the MAE of the MLP with a single hidden layer using the predicted PBL heights results in a smaller value compared to models not using the forecasted PBL heights, i.e., there is a decrease in MAE from 7.3918 ppb to 6.6540 ppb. Among all models, the range of decreases in MAE is from 1.63% (MLP with L = 1 at 6-085-0005) to 10.13% (MLP with L = 1 at 6-077-1002). For the RMSE metric, MLP with two hidden layers using the forecasted PBL heights yields 6.8828 ppb at station 6-19-4001, which is much smaller than that seen in models not using the predicted PBL heights, i.e., a decrease of 5.48%. The enhancement rates in terms of RMSE over all MLP-based models are from 1.70% (MLP with L = 1 at 6-085-0005) to 8.43% (MLP with L = 2 at 6-077-1002) by considering the forecasted PBL heights additionally. However, we observe that IOA has no significant change regardless of use of the forecasted PBL heights.

## 5. Conclusions

In this paper, we investigated the effectiveness of using forecasted planetary boundary layer (PBL) information obtained from WRF model outputs on the performance of our proposed measurement data-driven surface ozone prediction models. We used the meteorological data collected from air monitoring stations operated by the U.S. Environmental Protection Agent (EPA) as measurement data. We considered the North America Mesoscale (NAM) model, which is one of the regional weather forecast models run by the National Centers for Environmental Prediction (NCEP), to gather the predicted PBL data. We note that the NAM model forecasts hourly PBL heights up to 36 h every 6 h. We considered two machine learning algorithms for surface ozone forecasting models, the multilayer perceptron (MLP) and the bidirectional long short-term memory (LSTM) models. To apply the measurement data, as well as the predicted PBL height data, as inputs to these two forecasting models, we needed the following consideration: For the bidirectional LSTM-based model, a sequence-to-sequence structure was proposed because the measurement data and predicted PBL height data are in sequential relation, but they have a different number of features. That is, the measurement data considers three features, such as ozone, temperature, and relative humidity, while the predicted PBL height data have only one single feature with itself. To verify the effectiveness of using predicted PBL heights, we compared the performances of the MLP-based and bidirectional LSTM-based models where the historical measurement data and the forecasted PBL heights together were considered and compared with the models where only past measurement data were used, in terms of IOA, MAE, and RMSE. We showed that the MLP-based and bidirectional LSTM-based models that used the additional predicted PBL heights as an input yielded a performance enhancement, i.e., lower MAE and RMSE, compared to the models without the forecasted PBL heights. However, neither model showed a significant difference in IOA. Therefore, utilization of predicted PBL heights can improve the forecasting accuracy for surface ozone concentrations. In future work, we will study the effect of wind speed and NOx as well as predicted PBL heights on ozone forecasting, since wind speed and NOx are considered important features for ozone forecasting.

## Figures and Tables

**Table 1:** Information (latitude, longitude, and location name) of the considered ozone monitoring stations.

Station ID	Latitude	Longitude	City Name
6-019-4001	36.597442	−119.503659	Parlier
6-037-1103	34.066590	−118.226755	Los Angeles
6-037-1302	33.901389	−118.205000	Compton
6-065-0012	33.920860	−116.858410	Banning
6-065-8001	33.999580	−117.416010	Rubidoux
6-077-1002	37.950741	−121.268523	Stockton
6-085-0005	37.348497	−121.894898	San Jose

**Table 2:** Geographical information of the selected grid point for the NAM model corresponding to each ozone monitoring station.

Station ID	Latitude	Longitude	Distance [km]
6-019-4001	36.553	−119.54	5.91
6-037-1103	34.1193	−118.204	6.22
6-037-1302	33.9059	−118.159	4.27
6-065-0012	33.8712	−116.83	6.11
6-065-8001	34.0145	−117.387	3.15
6-077-1002	37.9313	−121.237	3.51
6-085-0005	37.3871	−121.926	5.10

**Table 3:** Performance comparison of the MLP-based and bidirectional LSTM-based surface ozone forecasting models with and without the forecasted PBL heights in terms of IOA, MAE, and RMSE.

Station ID	Method	Performance Metrics		
		IOA	MAE (ppb)	RMSE (ppb)
6-019-4001	MLP (L = 1, *w*/*o* PBL)	0.8979	6.0113	7.3830
	MLP (L = 2, *w*/*o* PBL)	0.8999	5.9413	7.2816
	LSTM (*w*/*o* PBL)	0.8910	6.1784	7.5812
	MLP (L = 1, *w*/PBL)	0.8949	5.6681	6.9280
	MLP (L = 2, *w*/PBL)	0.8981	5.6062	6.8828
	BLSTM (*w*/PBL)	0.8921	5.8521	7.1612
6-037-1103	MLP (L = 1, *w*/*o* PBL)	0.8567	6.2489	7.7356
	MLP (L = 2, *w*/*o* PBL)	0.8653	6.2117	7.7106
	LSTM (*w*/*o* PBL)	0.8566	6.4072	7.8248
	MLP (L = 1, *w*/PBL)	0.8728	5.8652	7.3019
	MLP (L = 2, *w*/PBL)	0.8713	5.8582	7.2794
	BLSTM (*w*/PBL)	0.8679	5.9817	7.3883
6-037-1302	MLP (L = 1, *w*/*o* PBL)	0.8452	6.3339	7.7883
	MLP (L = 2, *w*/*o* PBL)	0.8465	6.2905	7.7604
	LSTM (*w*/*o* PBL)	0.8405	6.5320	7.9609
	MLP (L = 1, *w*/PBL)	0.8459	6.1128	7.5829
	MLP (L = 2, *w*/PBL)	0.8490	6.0591	7.5255
	BLSTM (*w*/PBL)	0.8458	6.1965	7.7084
6-065-0012	MLP (L = 1, *w*/*o* PBL)	0.7951	7.8553	9.6385
	MLP (L = 2, *w*/*o* PBL)	0.7869	7.8205	9.5761
	LSTM (*w*/*o* PBL)	0.7814	8.1510	9.8979
	MLP (L = 1, *w*/PBL)	0.7968	7.5523	9.2330
	MLP (L = 2, *w*/PBL)	0.7950	7.5158	9.1958
	BLSTM (*w*/PBL)	0.7902	7.8221	9.5657
6-065-8001	MLP (L = 1, *w*/*o* PBL)	0.8794	7.3918	9.1394
	MLP (L = 2, *w*/*o* PBL)	0.8844	7.2922	9.0526
	LSTM (*w*/*o* PBL)	0.8801	7.5667	9.3660
	MLP (L = 1, *w*/PBL)	0.8901	6.6540	8.4441
	MLP (L = 2, *w*/PBL)	0.8920	6.6052	8.3415
	BLSTM (*w*/PBL)	0.8880	6.8128	8.5979
6-077-1002	MLP (L = 1, *w*/*o* PBL)	0.8444	5.9542	7.2909
	MLP (L = 2, *w*/*o* PBL)	0.8411	5.9044	7.2683
	LSTM (*w*/*o* PBL)	0.8300	6.3042	7.6390
	MLP (L = 1, *w*/PBL)	0.8686	5.3512	6.6830
	MLP (L = 2, *w*/PBL)	0.8671	5.3111	6.6556
	BLSTM (*w*/PBL)	0.8640	5.4996	6.9115
6-085-0005	MLP (L = 1, *w*/*o* PBL)	0.7917	6.1846	7.5674
	MLP (L = 2, *w*/*o* PBL)	0.8018	6.1317	7.5343
	LSTM (*w*/*o* PBL)	0.7890	6.4002	7.7771
	MLP (L = 1, *w*/PBL)	0.7893	6.0839	7.4389
	MLP (L = 2, *w*/PBL)	0.8033	6.0280	7.3890
	BLSTM (*w*/PBL)	0.7912	6.3062	7.6778

## Data Availability

Not applicable.
